# Fantastic Downy Mildew Pathogens and How to Find Them: Advances in Detection and Diagnostics

**DOI:** 10.3390/plants10030435

**Published:** 2021-02-25

**Authors:** Andres F. Salcedo, Savithri Purayannur, Jeffrey R. Standish, Timothy Miles, Lindsey Thiessen, Lina M. Quesada-Ocampo

**Affiliations:** 1 Department of Entomology and Plant Pathology, North Carolina State University, Raleigh, NC 27695-7613, USA; afsalced@ncsu.edu (A.F.S.); spuraya@ncsu.edu (S.P.); jrstandi@ncsu.edu (J.R.S.); ldthiess@ncsu.edu (L.T.); 2 Department of Plant, Soil and Microbial Sciences, Michigan State University, East Lansing, MI 48824, USA; milesti2@msu.edu

**Keywords:** downy mildews, molecular diagnostics, plant pathogens

## Abstract

Downy mildews affect important crops and cause severe losses in production worldwide. Accurate identification and monitoring of these plant pathogens, especially at early stages of the disease, is fundamental in achieving effective disease control. The rapid development of molecular methods for diagnosis has provided more specific, fast, reliable, sensitive, and portable alternatives for plant pathogen detection and quantification than traditional approaches. In this review, we provide information on the use of molecular markers, serological techniques, and nucleic acid amplification technologies for downy mildew diagnosis, highlighting the benefits and disadvantages of the technologies and target selection. We emphasize the importance of incorporating information on pathogen variability in virulence and fungicide resistance for disease management and how the development and application of diagnostic assays based on standard and promising technologies, including high-throughput sequencing and genomics, are revolutionizing the development of species-specific assays suitable for in-field diagnosis. Our review provides an overview of molecular detection technologies and a practical guide for selecting the best approaches for diagnosis.

## 1. Downy Mildew Pathogens and How to Find Them 

Downy mildew (DM) pathogens include several species of obligate oomycetes that can cause devastating damage to commercial [1], landscape [2], and natural ecosystem plants [3,4,5]. Species such as *Plasmopara viticola* [6], *Pseudoperonospora cubensis* [7], *Pseudoperonospora humuli* [8,9], *Peronospora belbahrii* [10], *Plasmopara obducens* [11], *Peronospora tabacina* [12] *Peronospora effusa* [13], *Peronosclerospora philippinensis*, and *Sclerophthora rayssiae* var. *zeae* [14] have resulted in significant losses due to downy mildew epidemics around the world. In some instances, the epidemics have been so severe that they have prompted historical shifts in crop production [15,16,17,18]. In addition to the aggressiveness of these pathogens, fungicide insensitivity further compounds losses attributed to disease [19,20,21,22]. Thus, research to improve diagnostics and management of downy mildew pathogens has become a priority for the scientific community in recent years [23,24,25,26].

**Figure 1:**
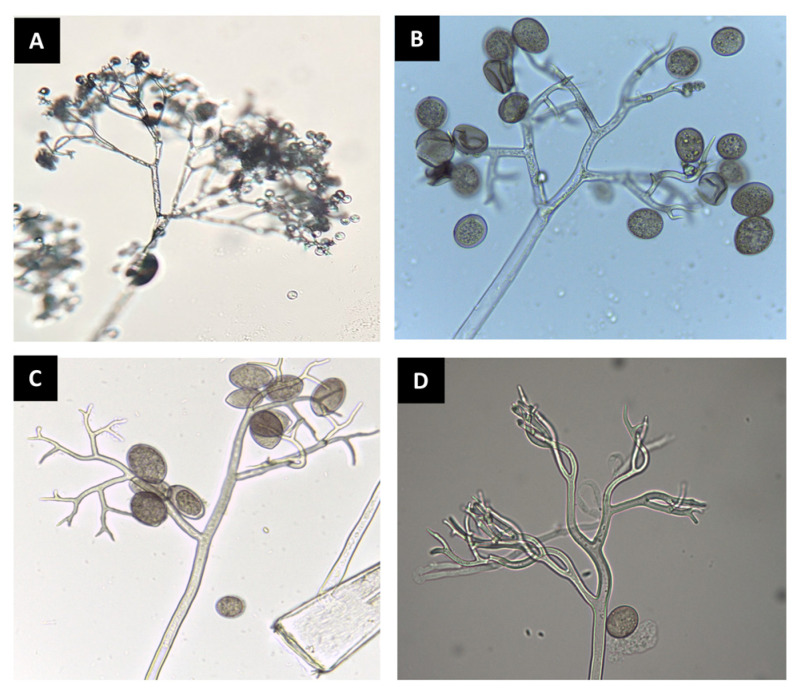
Sporangiophores and sporangia of downy mildew pathogens observed under a compound microscope. *Bremia lactucae* (**A**); *Peronospora belbahrii* (**B**); *Pseudoperonospora cubensis* (**C**); *Peronospora chenopodii-ambrosioidis* (**D**).

**Figure 2:**
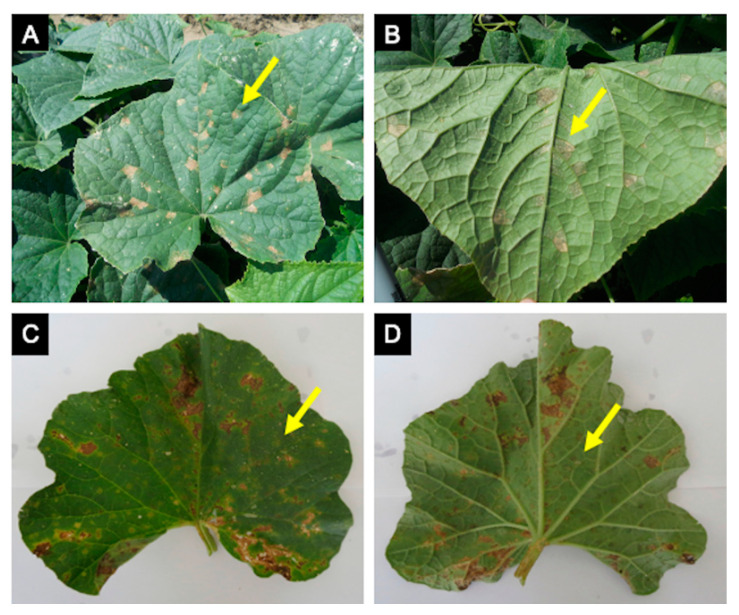
Cucurbit downy mildew caused by *Pseudoperonospora cubensis* in cucumber and cantaloupe. Cucumber symptoms (**A**) and signs (**B**) are very distinct, while cantaloupe symptoms (**C**) are often confused with other leaf spots or injury due to little sporulation on the underside of the leaf (**D**).

**Figure 3:**
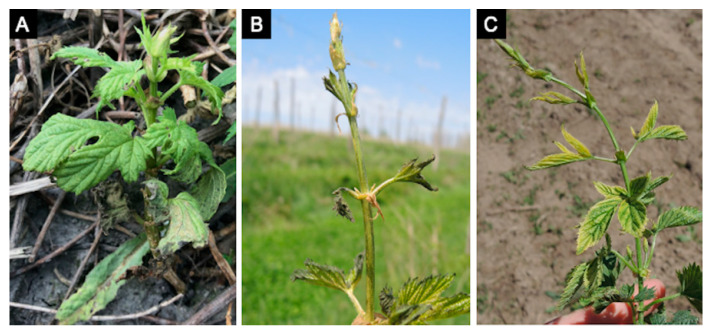
Hop downy mildew caused by *Pseudoperonospora humuli* sporulates profusely under humid conditions on abaxial surfaces of initial shoots in the spring called “primary spikes” (**A**) but these can be confused with abiotic damage such as frost injury (**B**) or herbicide damage like glyphosate injury (**C**). Panel photos B and C are courtesy E. Lizotte, Michigan State University Extension.

**Figure 4:**
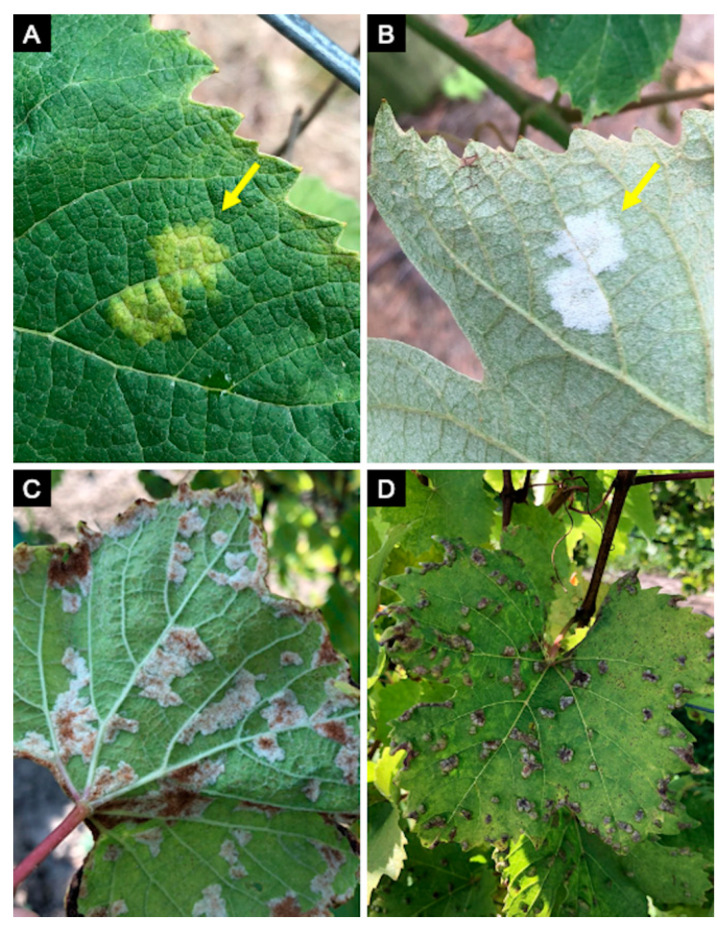
Grape downy mildew, caused by *Plasmopara viticola*, symptoms and signs on adaxial (**A**) and abaxial leaves (**B**) can be confused with grape erineum mite (*Colomerus vitis*) (**C**) when looking only at the abaxial side of the leaves. Grape erineum mite infections usually accompany gall structures on the adaxial surface which aids in diagnosis (**D**).

**Figure 5:**
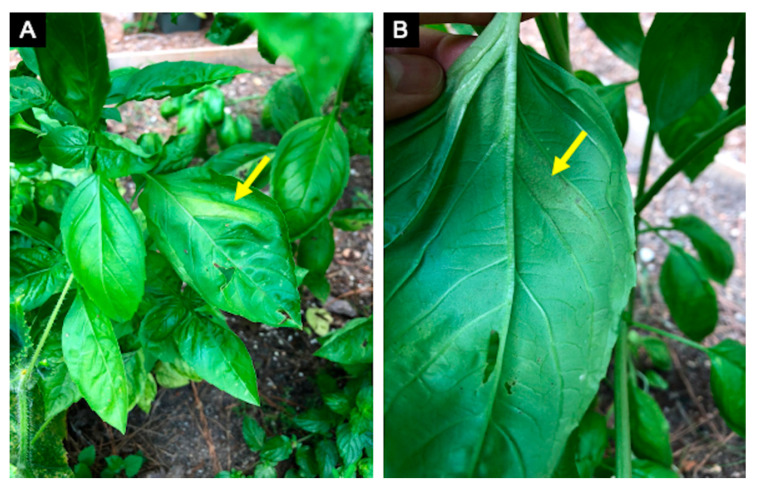
Basil downy mildew, caused by *Peronospora belbahrii*, symptoms (**A**) can be confused with nutritional disorders in the absence of sporulation on the underside of the leaf (**B**).

The diagnostics of downy mildew diseases has mainly relied upon direct observation of symptoms and signs using the naked eye or hand lenses and microscopes [27]. This is possible after observing their sexual (e.g., antheridia and oogonia) and asexual structures (e.g., sporangiophores, sporangia, and zoospores) (Figure 1) involved in survival and dispersion, and because many downy mildew pathogens produce distinctive foliar signs and symptoms when colonizing a host plant [2,28,29]. However, such methods fall short when detection in seed or planting material is needed [8,23], when symptoms and/or signs are not characteristic enough, resulting in misdiagnosis [30], or when the pathogen identity to species, pathotype, or clade level has disease management implications [31] (Figure 2, Figure 3, Figure 4 and Figure 5). For example, *Ps. humuli*, the downy mildew pathogen of hop, causes systemic infections including the rhizomes used as planting material to start new hop yards [8]. Similarly, the identification of basil downy mildew caused by *Pe. belbahrii* can be complicated because foliar lesions can be confused with nutritional disorders in the absence of pathogen sporulation [30]. Furthermore, isolates of *Ps. cubensis* causing downy mildew in cucurbits belong to two host-adapted clades [31], with clade 1 isolates mainly infecting watermelon, squash, and pumpkin, and clade 2 isolates mainly infecting cucumber and cantaloupe, thus initiation of fungicide applications in a particular cucurbit crop depends on the clade present in a given area and time during the growing season [31,32]. Molecular techniques can assist with early detection of the pathogen and provide more accurate diagnostics for timely deployment of disease management strategies for downy mildew [33,34].

The rapid identification of disease-causing organisms as well as disease forecasting to reduce the use of pesticides [35,36] have created the necessity to develop more rapid, sensitive, versatile, high-throughput, and cost-efficient markers to identify and quantify plant pathogens. Unfortunately, downy mildew pathogens have been under-represented in oomycete phylogenetic studies and marker development [37]. Visual vs. molecular approaches for downy mildew diagnostics have different advantages and disadvantages [34], but rapid and accurate diagnostics are needed because, under favorable weather conditions, a field or greenhouse infected with downy mildew can result in complete loss of the crop in just a few days [38,39,40]. On-site visual inspection of symptoms and signs may provide rapid diagnosis but requires trained personnel familiar with the particular downy mildew disease and the presence of distinct symptoms and/or signs [8,27,30]. Molecular diagnostic assays can be performed within a day but, in most cases, require a sample being taken to the laboratory, which can delay the process by several days [34]. This is also true when using conventional microscopy in the laboratory if on-site identification is not possible [41]. Molecular diagnostics can also provide additional information other than pathogen identity, such as fungicide sensitivity, virulence, aggressiveness, or pathogenicity, if such markers are available for a downy mildew pathogen [32,33]. However, for practical use of this information in disease management, results from assays need to be available quickly [41]. In this regard, field-deployable platforms for molecular diagnostics will be critical to unlock the potential for novel molecular markers and technologies to revolutionize disease management [42,43,44].

## 2. Advantages and Disadvantages of Different Marker Types

Visual-based diagnostic methods for plant pathogens are largely based on the observation of phenotypic characters and host range associations that require time and expertise for interpretation [45] and are frequently limited to taxonomic determination above species. By contrast, molecular techniques have changed the diagnostic emphasis towards pathogen presence and quantity using nucleic acids or proteins [46]. These techniques include serology (immunology), isoenzyme comparison, and nucleic acid-based technologies [44,46,47] (Table 1).

Serology methods rely on the use of specific (monoclonal) antibodies to detect their respective antigenic determinant or epitope, which can be cellular components or macromolecules like pathogen proteins, complex carbohydrates, polynucleotides, or lipopolysaccharides [46,48]. Antigen-specific antibody (immunoglobulin) is labeled with a fluorescent dye or enzyme that serves as a marker to detect and quantify the pathogen [49,50]. Labeling of specific antibodies has led to the development of sensitive methods like the enzyme-linked immunosorbent assay (ELISA) and immunochromatographic test strip (lateral flow assay), which has been used extensively for diagnosis given its speed, simplicity, and low cost. 

Oomycete monoclonal antibodies have been developed to detect zoospores, cysts, or cell wall proteins of *Pythium* [51,52] and *Phytophthora* [52,53,54] and are available as commercial kits [53]. For downy mildew pathogens, monoclonal antibodies have been produced to detect *Pe. Destructor*, the causal agent of onion downy mildew [54]. However, since the limit of detection is 500 sporangia, its potential use for biosurveillance has been questioned [55]. Lateral flow technology uses specific monoclonal antibodies against a pathogen that allows for a quick, cheap, and convenient diagnostic test that can be performed on-site for a fraction of the cost of an ELISA [56]. Lateral flow technology is sold as a prefabricated strip (immunostrips) containing an antibody marker that is activated with the presence of the pathogen in a sample [57]. However, one of the major limitations of this technology is the potential cross-reactivity with other oomycete species [54,55]. Immunostrips produced by Agdia Inc. (Elkhart, IN, USA) and Neogen Corp. (Lansing, MI, USA) are available for the detection of *Pythium* and *Phytophthora*, but such resources are unavailable for downy mildew pathogens, nonetheless, because of their versatility and ease of use, inmmunostrips have the potential to be one of the best diagnostic assays once the prohibitive cost of generating specific antibodies is reduced.

Isozymatic profiles (multiple forms of an enzyme that differ in size, shape, and charge) have also been used as a diagnostic method [50]. This approach has been applied to identify three species of maize downy mildew *Pernosclerospora* spp. [58], and in the genetic diversity evaluation of sunflower downy mildew *Pl. halstedii* [59]. Since isozyme analysis requires a large quantity of pathogen tissue, compared with serology, and is less reliable and informative than nucleic acid-based diagnosis, this approach is considered a complement to visual methods [50]. 

Nucleic acid-based detection methods rely on the variations in the nucleotide sequences of the pathogen’s nuclear and/or mitochondrial genome and can be used at any stage of pathogen development. They have become the most popular approach for diagnosis because they can be adaptable, quick, specific, sensitive, reliable, reproducible, and cost-effective, and can differentiate intraspecific taxonomic categories. These methods detect the presence of the pathogen with a molecular marker [60] and include classic approaches like restriction fragment length polymorphism (RFLP), amplified fragment length polymorphism (AFLP), random amplified polymorphic DNA (RAPD), micro- and minisatellites, which are still useful but require significant laboratory infrastructure and sufficient DNA for detection [61,62]. Microsatellites or simple sequence repeats (SSRs) markers have been helpful to distinguish isolates and to develop diagnostic markers for the sorghum downy mildew *Pe. sorghi* [63], and the characterization of *Ps. cubensis* populations [31,64]. The use of SSRs in diagnostics is limited and their development can be expensive and laborious, although, recently, SSRs identification and development are being accelerated by high-throughput sequencing (HTS) and bioinformatic tools [62].

The most prevalent method to test nucleic acid-based markers is polymerase chain reaction (PCR) followed by sequencing [44,45], and the most common locus/loci for molecular markers in oomycetes for phylogenetic studies and sequence-based identification have included the non-coding nuclear internal transcribed spacer region (ITS1-ITS2), D1/D2 region, 28S of the larger subunit of the nuclear ribosomal DNA (nrDNA), mitochondrial ribosomal DNA (mrDNA) such as *L10*, mitochondrial genes such as *cox1-2, nad1, rps10*, mitochondrial intergenic regions (mtIGS), and nuclear protein-encoding genes such as *hsp90, β-tubulin, ef1a, ypt1, nad 1* [61,65,66,67,68]. For diagnostic purposes, the ideal target for a molecular marker needs to be highly specific without orthologous copies and sensitive to detect low quantities of pathogens, be amplified by common primers, and generate a distinctive product [65]. Additionally, markers need to be validated using a robust panel including geographically diverse isolates, closely related species, and field samples to minimize the effect of technical differences, molecular techniques used, cross-reactivity, and non-target amplification with the environment (i.e., plants and other microorganisms) [34].

Selection of the best locus is the most complicated step for marker development [34], and it is based on the purpose (diagnosis, quantification, phylogenetic studies, barcoding, etc.) and molecular techniques used. The ITS has been considered as the universal DNA barcode (a short portion of the genome that is used to identify species through reference to DNA sequence libraries or databases) for fungi and oomycetes. The ITS regions have substantial sequence representation in databases, simplifying the identification by sequence similarity. In addition, ITS markers have shown consistency and reproducibility using both conventional PCR and quantitative PCR (qPCR) [34,35,69]. The ITS includes the ITS1 and ITS2 regions, separated by the 5.8S gene, and is situated between the 18S (SSU) and 28S (LSU) genes with more than 250 tandem repeats per cell, making the regions a desirable target when DNA quantity is low [68]. Since ITS regions are conserved loci, it is easy to have a panel of common PCR primers, which simplifies the obtention of PCR products for regular pathogen identification [69]. 

**Table 1:** Major advantages and disadvantages of molecular markers in the diagnosis of downy mildews.

Source	Loci/Type	Advantages	Disadvantages	Examples
**Nuclear**	**Ribosomal and Internal Transcribed Spacer (ITS)**	-High reproducibility -Abundant copies -Common primers	-Copy heterogeneity -Low resolution for cryptic species -Species cross-reactivity	*Pe destructor* [70] *Pe arborescens* [71] *Ps belbahrii* [72] *Plasmopara* spp. [64] *B.lactucae* [64] *Ps humuli* [73]
	**Housekeeping**	-Common primers -known genes	-Low polymorphisms and reproducibility -Limited for phylogenetic analysis	*Ps belbahrii* [26] *Ps cubensis* [74]
	**Species-specific**	-species-specific primers	-Low polymorphisms and reproducibility -Limited for phylogenetic analysis	*Ps humuli* [23] *Ps cubensis* [32,33]
	**Multilocus**	-Improves phylogenetic interpretation -Infraspecific resolution -High variability	-Low reproducibility -More labor	*Ps cubensis* [66] *Ps. humuli* [66].
**Mitochondrial**	**Single locus**	-Improves phylogenetic interpretation -Infraspecific resolution -High variability	-Uniparental heritance -Limited in detecting hybrid species	*B. lactucae* [75] *Ps cubensis* [76]
**Antigen**	**ELISA**	-Speed and simplicity -High-throughput	-Requires monoclonal antibodies -Species cross-reactivity -Limited use for biosurveillance	*Pe destructor* [54]
	**Immunostrips**	-Speed and simplicity -Cost-effective -Portability	-Requires monoclonal antibody -Species cross-reactivity	Still not available for downy mildew pathogens
**Enzymatic profile**	**Isozymes**	-Codominant markers -Complement to phenotypic data	-Large amount of tissue is required -Low polymorphisms -Influenced by the environment	*Pernosclerospora* spp. [58] *P. halstedii* [59]

ITS regions have been successfully used to develop qPCR assays for detecting *Pe. destructor* [70] and *Pe. arborescens* (opium poppy downy mildew) [71], and as markers for detecting the basil downy mildew pathogen *Pe. belbahrii* using conventional PCR [72]. However, ITS regions have significant heterogeneity among the repeats and indels that can produce ambiguity on sequence chromatograms or misalignments [77]. Some downy mildew pathogens of the *Plasmopara* genera and relatives carry large insertions, making amplification and sequencing of ITS challenging [78]. Moreover, ITS regions sometimes cannot differentiate between species or infraspecific taxonomic categories, creating a misdiagnosis by cross-reaction [79]. An ITS-based specific detection assay for *Pe. effusa* on spinach failed to discriminate it from the closely related *Pe. schachtii*, the causal agent of downy mildew on sugar beet [80]. Likewise, specific primers, designed in the ITS region, could not differentiate *Ps. cubensis* from sister species *Ps. humuli*, which initially was suggested as a synonymous species [73,81], but a multilocus analysis including both mitochondrial and nuclear protein-coding genes revealed that *Ps. cubensis* and *Ps. humuli* are distinct species [66,81].

The use of a single locus for developing species-specific markers provides a straightforward and safe way to identify and quantify pathogen propagules, avoiding cross-reactions. However, the use of a single conserved nuclear or housekeeping gene may not provide enough variability for species delimitation due to functional constraints and sequence identity [82]. Moreover, the development of species-specific molecular markers based on non-housekeeping protein-coding nuclear genes requires the screening and testing of a large set of candidate genes that contain enough levels of sequence polymorphism among closely related species [69]. Potential disadvantages of those loci are that they are usually a difficult target for PCR amplification [83], they are usually not informative for phylogenetic studies [84], and they may have lower reproducibility, making the DNA-based assays more prone to false positives [85].

Some major limitations of diagnostic molecular markers based on nrDNA and nuclear protein-encoding genes, including the lack of sensitivity to low pathogen levels and the inability to detect cryptic species [86], can be overcome by using mitochondrial loci as diagnostic markers. Mitochondrial loci have been considered ideal for both pathogen detection and diversity studies, because they have multiple copies like nrDNA, evolve rapidly, and provide abundant genotypic variability for the development of molecular markers to differentiate closely related species [87,88]. Mitochondrial DNA (mtDNA) has been used more frequently when developing markers for oomycetes than fungi since primer design is simpler because oomycete mitogenomes do not contain introns [86]. To date, the mitochondrial genomes of over 87 species of oomycetes, including several important genera like *Phytophthora*, *Pythium*, and a broad range of downy mildew pathogens, are available [82]. These genomic resources are useful for designing specific markers that focus on unique gene order, unique putative ORFs (open reading frames) or indels and avoid the dependency on PCR stringency while providing flexibility to develop assays for genus, species, or infraspecific taxonomic categories [32,75,82]. For diagnostic purposes, one of the best options is to combine mtDNA loci with qPCR to improve the detection of low pathogen levels in environmental samples and early stages of infection in the host [86]. This strategy allowed the development of species-specific markers based on the cytochrome oxidase 2 (*cox2*) gene for *Ps. cubensis* [76] and an mtDNA region for lettuce downy mildew *Bremia lactucae* [75].

However, the use of mitochondrial markers has some limitations: mtDNA is inherited uniparentally, as a single linkage group, creating conflicts with species trees constructed with other phylogenetic approaches since their estimates are not independent. Additionally, mtDNA would not represent the history of the pathogen populations by their complex mutation process, the phenomena of incomplete lineage sorting (retention and stochastic sorting of ancestral polymorphisms), and high allele extinction rate, since it has only a quarter of the population size of nuclear DNA (nDNA) [86,89,90]. Furthermore, mtDNA phylogenetic conclusions about the detection of hybrid species need rigorous additional nuclear marker assays [82]. Finally, it is unknown if mitochondrial copy number varies among species, isolates, or during different phases of infection, influencing the accuracy of quantification assays [61].

In oomycetes, the mitochondrial *cox1* is considered a universal barcode for identification of closely related species of *Phytophthora* and *Pythium* and a wide range of downy mildew pathogens using common primers [91]. However, *cox1* amplification efficiency and resolution power vary across different downy mildew lineages, which has led to *cox2* and *cox2-1* being suggested as better barcode because of their PCR performance and the improvement in infraspecific detection [87].

These shortcomings indicate that when a single locus fails to define species boundaries among closely related taxa, a reliable identification strategy will require a multilocus approach with other mitochondrial and nuclear genes [60,92,93]. Multilocus approaches have been effective in deciphering obscure phylogenetic relationships between different species of *Pythium* and *Phytophthora* [60,94,95] and at delimiting *Ps. cubensis* from *Ps. humuli* [66]. The availability of downy mildew pathogens pan-genomes and genome-scale phylogenetics [96] will allow the evaluation of multiple candidate loci for marker development using bioinformatics methods and the development of multiplexed PCR assays for accurate diagnosis [32].

The detection and identification of downy mildew pathogens are fundamental for disease management. Molecular diagnostic assays do not have most of the disadvantages associated with traditional detection methods, but the selection of a particular molecular assay is not free of caveats and depends on the main objective (detection, biosurveillance, pathogen relatedness, decision making on fungicide use, pathogen population structure, etc.). However, the development of more accurate DNA-based tests and better molecular markers for the detection of species and infraspecific taxonomic categories will continue as we increase the genetic and genomic information about downy mildew pathogens.

## 3. Advances in Genomics Provide New Opportunities and Challenges for Diagnostic Marker Development 

Recent advancements in HTS have revolutionized disease diagnostics in plant pathology. Technological advances and plummeting costs have made the sequencing of even non-culturable, non-model organisms like downy mildew pathogens possible [12,23,97,98]. Though whole genome sequencing (WGS) enables the discovery of unique loci that can be used to develop markers of high specificity for the detection of plant pathogens [23], technical difficulties associated with the pathogen culturing and isolation of DNA have complicated the sequencing of downy mildew pathogens. Sequencing efficiency depends largely on the purity of the isolated DNA and the proportion of the organism under study, which is difficult for biotrophic pathogens. An advantage of HTS technologies, however, is the possibility of using reduced representation and enrichment approaches to develop diagnostic markers even in the absence of a completely assembled genome [24,99]. In the grapevine downy mildew pathogen *Pl*. *viticola*, pyrosequencing-based random and microsatellite enrichment libraries enabled the discovery of 31 microsatellite markers [99]. Transcriptome sequencing of different isolates was used to identify diagnostic markers in the cucumber downy mildew pathogen *Ps*. *cubensis* [24]. Pathogen enrichment sequencing (PenSeq) is another enrichment method that can be used to identify polymorphisms in downy mildew pathogens. PenSeq is a method that uses prior knowledge on specific rapidly evolving sequences like oomycete effectors to design baits to sequence target regions. Though PenSeq has not been used for downy mildew diagnostics yet, the method has been successfully used to study population genomics in *P. infestans* [100] and *Albugo candida* [101]. It is interesting to note that Jouet et al. [101] used PenSeq to assess the microbial diversity of field samples by designing baits for 49 microbial species. PenSeq was shown to detect the presence and quantify specific pathogens in the authors’ samples [101]. These examples indicate the practicality of using enrichment and complexity reduction methods in circumventing the difficulties associated with next-generation sequencing-based detection of obligate pathogens like those that cause downy mildews.

In recent years, the genomes of many downy mildew pathogens have been sequenced [6,12,23,92,97]. Though microsatellites have been discovered in sequenced genomes [92], the biotrophic nature of these pathogens have complicated the use of sequencing data for the development of markers of high specificity. The presence of contaminating sequences from the host and other microorganisms is one challenge associated with diagnostic marker development in downy mildew pathogens [33]. The alignment of assembled contigs against public databases and filtering sequences that show similarity to bacterial and host sequences is a simple method to remove contaminant sequences, and it has been used to clean the *Pl. muralis* genome using customized pipelines [6]. This approach, however, may reduce the quality of the genome assembly in heavily contaminated samples. Processing sequenced reads before assembly through taxonomic filtering tools may help in sieving out potential contaminating sequences without compromising assembly quality [23,37]. Contaminating sequences have been removed from the *Ps. humuli* genome [23] using GC proportion and similarity to sequences in public databases to separate a preliminary assembly into distinct taxa using the Blobology tool [102]. A similar approach based on metagenomic filtering using the Contig Annotation Tool (CAT) removed contaminating sequences from the *Pe. effusa* genome [37], generating an assembly comparable to those of pathogens that can be isolated in axenic culture (Figure 5). Rahman et al. [23] used the Blobology tool on Illumina reads to filter contaminants from the *Ps. humuli* genome while the Contig Annotation Tool was used on reads of *Pe. effusa* derived from a PacBio sequencer [37]. This suggests the adaptability of such filtering tools to long and 

**Figure 6:**
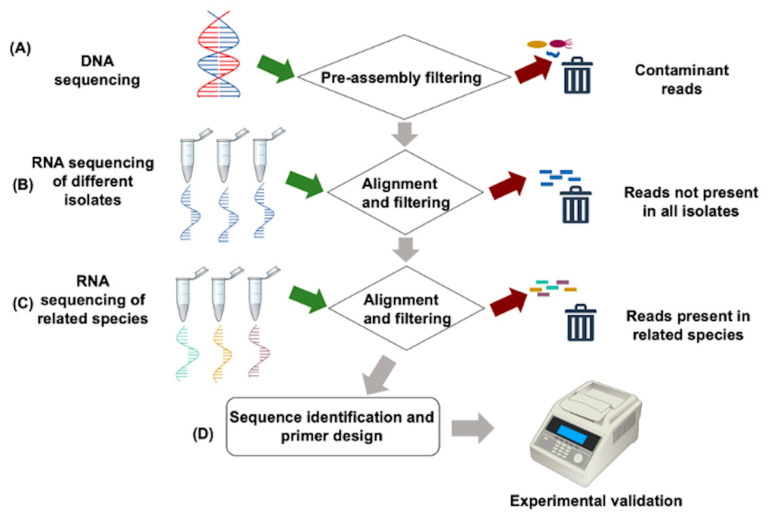
Example pipeline for identifying species-specific markers for downy mildew diagnostics using high-throughput sequencing (HTS). Pathogen DNA is sequenced and passed through a pre-assembly filtration tool to remove contaminant reads (**A**). Simultaneously, RNA of different isolates of the same pathogen are sequenced, aligned to the contaminant-free assembly, and reads that are not present in all the isolates are filtered out (**B**). Next, the reads that are present in the transcriptome of related species are filtered out (**C**). Finally, sequences of species-specific genes are identified and are experimentally validated through polymerase chain reaction (PCR) (**D**).

Many of the disadvantages presented by standard loci for developing markers for diagnosis can be circumvented by comparative genomic analysis using HTS data to discover species-specific genomic regions or genes between highly similar downy mildew pathogen genomes at relatively low cost [34,65,69]. Comparisons of whole genomes or genomic fractions can be used to identify unique markers that can be evaluated in multiple isolates [93]. This strategy has been successfully applied to develop species-specific markers for the closely related species *Ps. cubensis* [24] and *Ps. humuli* [23] by using unique lineage-specific regions with transcript evidence in different isolates of one species but absent in the isolates of closely related species. These regions, which can be exons or complete genes of varying functional annotations, can be used for the development of presence–absence variation or amplicon size polymorphism-based DNA markers. Moreover, potential protein-specific differences can be exploited for the development of serological-based diagnostics once monoclonal antibodies are developed for specific downy mildew pathogen proteins [24]. This approach is conceivable, since the biotrophic lifestyle of downy mildew pathogens has lineage-specific metabolic adaptations, gene content, and enzyme function that contribute to fitness and host adaptation [103,104]. The examples of *Ps*. *cubensis* [24] and *Ps*. *humuli* [23] emphasize the importance of developing downy mildew-specific pipelines for the development of HTS-based species-specific diagnostic markers (Figure 6). An important point to consider is the existence of different pathotypes and clades in some downy mildew pathogens, as has been reported in *Pl*. *halstedii* and *Ps*. *cubensis*. It is therefore a good practice to experimentally validate markers developed through HTS-based pipelines in different isolates of the pathogen to ensure specificity [23]. 

## 4. Beyond Pathogen Detection: Diagnostics for Pathogen-Informed Management 

Due to their obligate nature, pathogens that cause downy mildew exhibit host-adaptation at the plant family, species, or genotype level that results in differences in aggressiveness, virulence, and/or pathogenicity [6,31]. Because there is little knowledge of the genetic basis of virulence in most host–downy mildew pathogen pathosystems, categories such as races and/or pathotypes have typically been used to describe host–pathogen interaction differences [105,106,107]. Races and pathotypes have been useful to describe isolate virulence and establish a nomenclature for resistance loci on the plant side to a particular downy mildew pathogen [108,109,110,111]. In *B. lactucae*, race and differential screenings are regularly performed when varieties are released in several markets at both public and private diagnostic laboratories (e.g., Eurofins Biodiagnostics, USA; Trical diagnostics, USA). However, across other downy mildew pathosystems, race and pathotype information has been of limited use for disease management because testing isolates on a set of host differentials is a cumbersome and slow process, true-to-type host differentials are not always available, and false negatives due to an escaped infection are common in obligate pathogens [7,34]. 

The development of virulence diagnostic assays remains a priority to incorporate virulence information into disease management of downy mildews [33,34]. Pathotypes of *Pl. halstedii* have been differentiated by using competitive allele-specific (KASP) PCR markers detecting polymorphisms in pathogen effectors [112]. Because *Pl. halstedii* is homothallic and forms oospores that can persist in soil, having a rapid pathotype diagnostic assay to test soilborne inoculum in infested fields can now inform disease resistance deployment efforts in sunflowers [112]. Clade-specific qPCR diagnostic markers can be used to determine the risk of cucurbit downy mildew infection in a particular cucurbit crop in combination with spore trapping [31,32]. These efforts of in-season virulence biosurveillance of downy mildew pathogens can not only inform host resistance deployment but also result in reduced fungicide applications by giving producers variety- and/or crop-specific recommendations [32,33]. 

Understanding effector function remains critical for the development of durable resistant varieties and monitoring effector presence in pathogen populations via diagnostics may provide valuable epidemiological information for disease management [112]. Core RXLR effectors in *Pl. halstedii* expressed in several pathotypes during infection in sunflower and potentially targeting broad-spectrum resistance have been identified [113]. A combination of transient effector expression in sunflower to characterize effector-induced hypersensitive responses (HRs) and sunflower mapping populations allowed the physical mapping of a sunflower resistance gene and established an avirulence/resistance gene pair target for breeding [113]. Efforts to use effectors for pathogen-assisted breeding have also been initiated in hop and cucurbits. Transcriptome analysis during infection on hop and across several isolates of *Ps. humuli* resulted in the identification of a core effectorome for this downy mildew [114]. Similarly, clade-specific and species-specific core effectors have been identified in *Ps. cubensis* [115]. Transient effector expression *in planta*, genetic mapping, or resistance gene enrichment sequencing (RenSeq) approaches can later be used to identify susceptibility or resistance genes [113,116]. Once effector–host gene pairs are identified, effector-based markers can be used to monitor pathogen populations for shifts in virulence via biosurveillance and help prevent serious outbreaks due to the introduction of virulent pathogen genotypes [33,117].

Along with cultural practices, chemical fungicides play an important role in managing downy mildews. In sensitive high-value crops such as cucurbits and grapes, prophylactic applications are often required to keep plant tissue protected prior to symptom development and this can drive the development of fungicide resistance [118,119]. There are several broad-spectrum fungicides that can be utilized, such as Captan, ethylene bisdithiocarbamates, oil-based and copper-based products. However, the use of these chemicals is becoming increasingly restricted and for smaller crops, such as hops, some of these products have never been available [20]. This has caused a shift within the chemical industry to more site-specific products that target specific cellular processes within oomycetes and disrupt their function (Table 2). To help stakeholders organize these chemicals and to prevent the development of fungicide resistance, these products have been grouped by specific target and mode of action. This is annually compiled by the Fungicide Resistance Action Committee (FRAC) and the most effective active ingredients against downy mildew pathogens belong to FRAC codes 4, 11, 21, 22, 27, 28, 29, 40, 43, 45, 49, and U17.

Fungicide resistance within several important site-specific products is widespread and well-studied within oomycetes. For example, quinone outside inhibitors (FRAC 11) target the *cytochrome B* gene and a well-characterized mutation has been denoted G143A. This mutation is a qualitative form of resistance in several fungi and oomycetes [120]. Other active ingredients that belong to FRAC 40 (i.e., Mandipropamid and Dimethomorph) are the backbone of several management programs in grapes and hops. In the grape downy mildew pathogen *Pl. viticola*, resistance has been heavily linked to modifications in codon 1105 in *cesA3* (a cellulose synthase gene) causing an isolates’ EC_50_ value to shift from <0.2 μg/mL to >240 μg/mL of mandipropamid [121,122]. In *Ps. cubensis*, a similar mutation has been noted in North Carolina and was particularly present in cucumber isolates [33]. Other mutations for various FRAC codes have been characterized in FRAC 21, 22, 45, and 49 in specific oomycete species as well [123,124,125,126] (Table 2). For other FRAC codes such as phenylamides (FRAC 4), resistance has been reported for nearly 40 years [127] but it is still not entirely clear how resistance might develop in downy mildew pathogens [128,129,130]. For certain FRAC codes (i.e., FRAC codes 27, 28, 29, and 43), resistance has been reported using detached leaf assays or by observations of field control failures, but no link to the molecular mechanisms of fungicide resistance has been reported [131].

**Table 2:** Site-specific fungicides commonly used to control downy mildews sorted by Fungicide Resistance Action Committee (FRAC) groups. Information related to active ingredient(s), mode of action, and molecular mechanism for resistance and oomycetes in which it has been identified is shown.

Active Ingredient	FRAC	Target Site	Resistance in Oomycetes	Molecular Mechanisms Known?	Downy Mildews Where Molecular Mechanisms Have Been Identified
Mefenoxam	4	RNA polymerase I	Yes [127]	In *Phytophthora infestans* but not in a downy mildew pathogen [128,129,130].	N/A
Famoxadone and fenamidone	11	Complex III: cytochrome bc1 (ubiquinol oxidase) at Qo site (cyt b gene)	Yes, [33,120]	Yes, G143A amino acid change in cyt b gene	*Pl. viticola* *Ps. cubensis*
Cyazofamid	21	Complex III: cytochrome bc1 (ubiquinone reductase) at Qi site	Yes [126]	Yes, in *Pl. viticola* an insertion in cyt B gene (i.e., E203-DE-V204)	*Pl. viticola*
Ethaboxam and zoxamide	22	β-tubulin	Yes [123]	Yes, C239S amino acid change in β-tubulin gene	*Pl. viticola*
Cymoxanil	27	Unknown	Yes [132]	Unknown	N/A
Propamocarb	28	Cell membrane permeability, fatty acids (proposed)	Yes [131]	Unknown	N/A
Fluazinam	29	Uncouplers of oxidative phosphorylation	Yes [133]	Unknown	N/A
Dimethomorph and mandipropamid	40	Cellulose synthase	Yes [33,124]	Yes, G1105W/V/S amino acid change in cellulose synthase gene (cesA3)	*Pl. viticola* *Ps. cubensis*
Fluopicolide	43	Delocalization of spectrin-like proteins	Yes [131]	Unknown	N/A
Ametoctradin	45	Complex III: cytochrome bc1 (ubiquinone reductase) at Qo site, stigmatellin-binding subsite	Yes [134]	Yes, S34L amino acid change in *Pl. viticola* in the cyt B gene	*Pl. viticola*
Oxathiapiprolin and fluoxapiprolin	49	Lipid homeostasis and transfer/storage	Yes [125]	Yes, G769W amino acid change in PcORP1 gene in *Phytophthora capsici*	N/A
Picarbutrazox	U17	Unknown	Not officially reported	Not found	N/A

Downy mildew pathogens are not culturable in the traditional sense, so coupling our understanding of the molecular mechanisms with current detection strategies has the potential to be informative for disease management. Conducting traditional assays for fungicide resistance testing in downy mildew pathogens is very laborious, so once specific alleles associated with resistance are identified, molecular assays can be used to discriminate them. These molecular assays can aid the biosurveillance of downy mildews by improving pathogen detection of (1) samples where early detection is critical for management, (2) samples with mixed pathogen genotypes that can be quickly discriminated, and (3) environmental spore monitoring/trapping. Fungicide resistance molecular assays have not been widely used to monitor downy mildew pathogens, but there are examples in powdery mildew pathogens. In a recent study of grapevine powdery mildew caused by the fungus *E. necator*, a TaqMan assay for G143A (FRAC 11 resistance) was able to be directly utilized on environmental grape leaf samples when isolate establishment was difficult to observe and the assay was able to detect these alleles on spore trap rods when very small quantities of spores were present (between five and 172 conidia) [135]. 

In that study, a few different methodologies were employed, including TaqMan allelic discrimination and digital droplet PCR using dual quenched probes. That said, if a single nucleotide polymorphism is involved in resistance, a number of additional PCR-based tools could be utilized, such as high-resolution melt curve analysis (HRM), KASP, and rhAmp SNP (RNAseH dependent PCR) [76,136]. All of these strategies have advantages and disadvantages when it comes to sensitivity, specificity, and their ability to handle a mixed sample. The advantage of HRM is that it can be easier and cheaper to implement because probes are not required. KASP, TaqMan, and rhAmp SNP tools are likely more specific and allow increased accuracy when identifying an SNP. These tools are also likely improved in their ability to handle a mixed sample where more than one allele is present. Digital droplet PCR is likely the most sensitive technique for analyzing mixed samples, but the technology can be restrictive. For example, the optics sometimes bleed from one channel to another and proprietary master mixes may be needed in order to generate specific oil droplets. 

## 5. Lab and Field-Deployable Platforms for Downy Mildew Diagnostics and Early Detection

Monitoring the concentrations of plant pathogen propagules can provide early warning to prevent disease epidemics by guiding the timing of preventive fungicide applications [137]. In some pathosystems, commercial companies have developed assays to detect airborne pathogens and inform management decisions (e.g., Revolution Crop Consultants, LLC, USA; Rothamsted Research, UK). Volumetric or impaction style spore traps have been successfully utilized to detect visually the airborne inoculum of several downy mildew pathogens prior to disease development, including *Pe. effusa*, *Pe. schachtii*, *Pe. destructor*, *Ps. humuli*, *Ps. cubensis*, and *B. lactucae* [31,32,39,40,75,138,139,140,141], but the process has some important drawbacks. Considerable time and training are required to process spore trap samples and to accurately identify the organism in question [73,142]. Instead, using molecular diagnostic markers to identify and/or quantify airborne sporangia in spore trap samples may provide greater speed, accuracy, and precision in pathogen identification.

This strategy has been used to successfully identify the airborne inoculum of several downy mildew pathogens prior to disease development. DNA from sporangia of *Pe. effusa* and *Pe. schachtii*, the cause of spinach and beet downy mildews, respectively, could be detected in spore trap samples and quantified using a marker based upon an SNP in the 18S ribosomal DNA [80]. A follow-up study using these markers identified that *Pe. effusa* sporangia were ever-present in the air currents of the Salinas Valley (California, U.S.A.) but suggested that sporangia volume in spore trap samples (as DNA copies) could predict disease incidence up to nine days prior [40]. In the case of the onion downy mildew pathogen, *Pe. destructor*, a TaqMan probe was developed based on ITS sequences [70]. This assay has a detection limit of one sporangium per m^3^ of air and was used with roto-rod spore samplers to identify sporangia between 5 and 15 days before symptoms were observed [70]. Airborne sporangia of the hop downy mildew pathogen, *Ps. humuli*, were detected in spore trap samples using primers based on ITS sequences that were specific to both *Ps. humuli* and the cucurbit downy mildew pathogen, *Ps. cubensis* [73]. Similar to the previously discussed results [80], this assay exhibits cross-reactivity and in this case cannot be deployed reliably in areas where both hop and cucurbits are grown [73]. However, early detection with this assay did help to improve hop downy mildew control or reduce fungicide inputs compared with the standard practices in four of six commercial hop yards during validation [73]. In contrast, a qPCR assay based on an SNP in a mitochondrial marker (*cox2* gene) was developed to differentiate between *Ps. humuli* and *Ps. cubensis* [76]. The probes used in that assay successfully identified isolates of both species as well as infected plant material with or without sporulation and successfully identified both species in spore trap samples [76]. As previously mentioned, isolates of *Ps. cubensis* belong to two clades that prefer either watermelon, squash, and pumpkin (clade 1), or cucumber and cantaloupe (clade 2) [31,32]. As part of a biosurveillance program, the clade-specific nuclear marker c2555.3e7 [24] was developed for use in a multiplex qPCR assay that successfully identified and quantified airborne sporangia of the two respective *Ps. cubensis* clades [32]. The marker accurately estimated inoculum load over a 2-year study and provided between 2 and 4 days of warning before symptoms developed on watermelon and cucumber, respectively [32]. Finally, a TaqMan assay based on mitochondrial DNA was developed for the detection of the lettuce downy mildew pathogen, *B. lactucae*, in spore trap samples [75]. This assay was highly specific and identified as little as a single sporangium, which enabled the detection of the pathogen during lettuce-free periods [75]. Trapping systems were then deployed in experimental plots established in commercial lettuce fields and fungicide applications were advised based upon a qPCR cycle threshold (Cq value) of 24, which was equivalent to 1136 sporangia or approximately 8.5 sporangia per m^3^ of air [143]. In three experiments, the advisory system reduced inputs by one, one, and three fungicide applications in each of the three experiments, respectively, and did not perform significantly differently from the standard calendar-based schedule; however, disease incidence was reduced by 50% when compared with the untreated control plot [143].

On a more limited basis, molecular-based diagnostic markers have also been used to identify and/or quantify downy mildew inoculum in seed batches and soil samples. For the basil and opium poppy downy mildews, primary infections of *Pe. belbahrii* and *Pe. arborescens* are believed to occur through contaminated seed stocks [72,144,145,146]. Species-specific ITS primers were developed to detect *Pe. belbahrii* propagules on basil seed and in plant tissue samples [144]. When validated, this assay amplified approximately 3 pg of *Pe. belbahrii* DNA (equal to one sporangium) per basil seed; the assay also detected systemic growth of the pathogen throughout symptomless leaves and stem sections [72]. Likewise, an ITS-based qPCR protocol was able to detect as little as 1.2 pg of *Pe. arborescens* DNA per microgram of opium poppy seed in commercial seed stocks [71]. Conversely, certain hosts of downy mildew pathogens are not seeded directly; rather, a specific organ is planted (e.g., onion bulbs and hop rhizomes) and knowledge of soilborne inoculum is paramount. The previously described ITS-based assay used to detect *Ps. humuli* in Oregon hop yards was also able to detect the DNA equivalent of 10 sporangia in 25 mg soil samples in nine out of 10 reactions [73]. Similarly, for *Pe. destructor*, the TaqMan probe assay described previously was also able to detect DNA equivalent to fewer than 10 sporangia (as a proxy for oospores and other propagules) per gram of dry soil [70].

The examples presented above all share a similar disadvantage: each system requires transportation of samples from the field to a laboratory. Further development of in-field disease detection systems may further reduce the time from sampling to identification and quantification of pathogens [147,148], and potentially allow for non-experts to process individual samples [142]. Isothermal amplification systems like loop-mediated isothermal amplification (LAMP) or recombinase polymerase amplification (RPA) allow for inexpensive heating block or water bath systems to be used as opposed to more expensive thermal cycling devices [149,150]. Portable thermocyclers for qPCR are also available (e.g., Biomeme, Quantbio), but assay cost considerations may prevent use in some cropping systems [55].

Loop-mediated isothermal amplification (LAMP) assays have been developed for in-field applications because assays can use crudely extracted DNA, reactions are rapid (within 30 min) using inexpensive hot water baths (60 °C), and products may be visualized with the unaided eye [150,151]. For grape downy mildew (*Pl. viticola*), a LAMP assay was developed for the rapid detection of inoculum [44] and demonstrated the ability for isothermal assays to be utilized in downy mildew systems. Further improvements include the development of quantitative LAMP assays using either a probe system [152] or various dyes [153,154,155] that have allowed for the development of portable, quantitative LAMP devices (e.g., Genie II, YouDoBio, Denmark; Bioranger, Diagenetix Inc., USA) to allow for rapid in-field quantification of target organisms. Unfortunately, LAMP is prone to contamination by the large concatenated amplicon products generated during amplification [156], and sensitivity issues when low concentrations of target DNA are assayed [157]. Furthermore, while LAMP is purported to have fewer issues with inhibitors [158,159], LAMP is sensitive to different amplification inhibitors [160], which may limit utility where crude in-field DNA extraction processes are needed.

Portable qPCR systems allow for use of previously developed lab assays in field environments or in labs with limited equipment. Several have been developed in animal and human systems [161,162] and may be easily adaptable to downy mildew markers that are already available [33,71,72]. Because inhibitors from crude DNA extractions are of concern to qPCR assay success, high-quality DNA extractions that remove inhibitors are necessary for accurate quantification [158,159,160]. Several rapid extraction techniques are available as kits commercially (e.g., M1 Sample Prep, Biomeme Inc.) and others have been developed that may be easily adapted to in-field assays [163,164].

Recombinase polymerase amplification (RPA) has been utilized to detect several oomycete species but, to the authors’ knowledge, has not been employed for downy mildew detection. The design of RPA assays is similar to qPCR and utilizes primer pairs that are 25−35 base pairs long each and a heavily modified probe depending on the platform (i.e., fluorometric or lateral flow). Like for LAMP-based techniques, detection occurs within 30 min and assays are very tolerant of inhibitors [165]. Customizable kits are commercially available by Twistdx Limited (Maidenhead, UK) and Agdia Inc. (Elkhart, IN, USA), which include lyophilized enzymes, other reagents, rehydration buffers, and MgSO4, which initiates the reaction. An adapted RPA assay based on the *trnM-trnP-trnM* mtDNA loci was able to detect 140 *Phytophthora* species even at low DNA concentrations (i.e., < 1 pg) [165]. A different mitochondrial locus known as the *atp9-nad9* region was also used for several species-specific detection assays for *P. sojae*, *P. sansomeana*, *P. ramorum*, and *P. kernoviae*, modifying the concentrations of the forward and the reverse primers [135,166]. Similarly, early detection of *P. infestans* on potato leaves was obtained experimentally by inoculation of detached leaves and an ITS-based RPA assay [149]. The authors detected the presence of *P. infestans* on potato leaves as early as three days after inoculation in these experiments.

Using RPA to detect downy mildew species has not been a focus of many research efforts, but RPA assays would likely be helpful if infections were systemic (e.g., hop downy mildew), in seed lots (e.g., downy mildew of spinach and basil), or when sampling environmental samples where inhibitors might be present (e.g., impaction traps) [34]. RPA reactions in most plant pathology research studies have utilized a semi-quantitative fluorometric approach. For fluorometric assays, several devices are available, including a T16 isothermal reader (Axxin, Fairfield, Australia), AmpliFire (Agdia Inc.), Smart-Dart or Bioranger (Diagenetix, Honolulu, HI), Genie II or III (Optigene, Horsham, UK), or a standard qPCR machine. It should be noted that preformulated kits that are commercially available use both the fluorometric and the lateral flow device method to make detection easier for the user but are less quantitative.

As stated above, species-specific molecular diagnostic markers have the potential to improve the speed and accuracy of downy mildew pathogen detection when used in conjunction with spore trap samples or when developed into field-friendly LAMP and RPA-based tests. Furthermore, the sensitivity of these markers allows for more efficient disease management strategies to be developed. Specific strategies include improved timing of fungicide applications, as observed with *B. lactucae* [144], or host-specific fungicide applications, as observed with the two respective host-adapted clades of *Ps. cubensis* [31,32].

## 6. Future Prospects: Portable Sequencing for Diagnostics and Biosurveillance

Accurate and timely diagnosis is key for controlling downy mildew pathogens. The biggest challenge in diagnostics is the discovery of specific markers that can be deployed in an efficient and cost-effective manner. As discussed previously, detection and quantification of the pathogen, even before the onset of visible symptoms and signs, is important for the deployment of management strategies and this has been possible with molecular techniques. However, molecular diagnosis is a two-step process, with there being spatial and temporal separation between sample collection in the field and molecular detection in the laboratory [34]. For this reason, portable machines that can accurately detect the presence of the pathogen in real time are the future of diagnostics. Until recently, genome sequencing had been confined to specialized laboratories and facilities because of the high cost associated with the technology and the technical expertise required for its execution. In the past few years, however, the availability of portable sequencing machines, like the MinION platform from Oxford Nanopore, has opened the possibility of real-time sequencing and diagnostics in plant pathology. With proven efficiency in even extreme environments such as Antarctica [167] and the International Space Station [168], MinION is a handheld sequencing device that can sequence large quantities of DNA in a short period of time with minimal hardware and software requirements. The MinION platform has been successfully used to diagnose various plant diseases [169,170]. Though it can be assumed that diagnosing complex oomycetes like the downy mildew pathogens would be trickier, MinION has proven its worth in diagnosing the biotrophic fungal pathogen *Puccinia striiformis* f.sp. *tritici* (*Pst*) [171]. An advantage of MinION is its ability to go beyond species-level diagnostics as a potential surveillance tool to analyze distinguishing features such as fungicide resistance and differential virulence at the race level in a population, as demonstrated for *Puccinia striiformis* f.sp. *tritici* (*Pst*) [171]. This holds immense promise in downy mildew diagnostics, especially in cases like *Ps*. *cubensis*, where multiple pathotypes/clades are reported, to diagnose in real time the dynamics of a population for the effective deployment of management strategies. Arguably, nanopore sequencing can also be used to select disease-free planting material like in the case of hop downy mildew, where systemically infected crowns can cause outbreaks in newly established hop yards.

Despite its many advantages, nanopore sequencing as a possible portable pathogen detection platform is not without its drawbacks. A potential challenge for the practical application of portable sequencing is in-field DNA isolation techniques. Though methods like magnetic bead-based extraction [171] and enzyme-based PDQeX extraction [172] have been used for fungi and viruses, respectively, their application in the DNA extraction of downy mildew pathogens remains to be tested. Another potential disadvantage with sequencing-based diagnosis using nanopore sequencing could be the overwhelming presence of host sequence that may mask pathogen DNA. However, nanopore sequencers can selectively sequence target regions in real time by rejecting undesirable sequences based on software-specified configurations, a feature that can be used to selectively amplify pathogen-specific sequences. Though the advantages of nanopore sequencing seem to outweigh the disadvantages in theory, the practicality of using the platform for in-field downy mildew diagnostics needs to be tested. Once established, nanopore sequencing could revolutionize in-field downy mildew diagnostics.

Other molecular detection technologies like isothermal amplification (e.g., LAMP and RPA) and new forms of CRISPR-*Cas*-based systems also show lots of potential to diagnose oomycetes [135,157,166,173,174]. These systems use a variety of unique chemistry approaches and can target both DNA and RNA templates. While the chemical techniques have been available for a number of years, studies are still being published which compare the merits of various isothermal approaches over conventional PCR-based methods [149,175]. In some situations, isothermal techniques have been deployed widely around the globe with minimal to no machine requirements [176]. CRISPR-*Cas*-based diagnostic systems are relatively newer and often require an initial amplification step. Primarily, their utility has been focused on plant viruses in the plant pathology community [174]. Significant medical research has been poured into these approaches because of recent viral disease outbreaks in humans (e.g., COVID-19, HPV, Zika, and dengue) and hopefully this will significantly aid other fields like plant pathology [177,178,179].

While morphological features of downy mildew pathogens are identifiable by trained experts, using molecular inoculum detection tools represents a diagnostic tool to identify downy mildews with high specificity. The molecular tools available range from expensive technical formats with the ability to quantitate rapidly (e.g., qPCR) to handheld devices for in-field detection (e.g., LAMP, RPA), which give diverse options for finding downy mildews. Furthermore, given the potential for genetic differences informing management decisions (e.g., fungicide sensitivity of species or clades), molecular inoculum detection tools can be used for fungicide timing and selection by non-expert users. Ultimately, the goal of these approaches is to bring the diagnostic tool closer to the point of care with a tool that is simple, quick, and highly accurate that can detect and diagnose diseases. Future research in these areas should allow us to more accurately find these fantastic downy mildews.

## Data Availability

Not applicable.
